# PREPARING FOR OLDER ADULTS TO AGE IN PLACE: COMMUNITY-BASED EFFORTS IN TAIWAN

**DOI:** 10.1093/geroni/igad104.0743

**Published:** 2023-12-21

## Abstract

Taiwan’s baby-boomers are about to join the super aged society and an action plan was called to increase public awareness of aging-in-place among all community-based senior service sites. A book, entitled How to Live with a Good Life and consisted of 25 topics, was published to guide older adults to prepare themselves in five domains: Healthy living, Social Participation, Recreation and Technology, Health and Long-term care, and Finance and Legal Protection. The purpose of this paper was to test how well the older adults (N=180) responded to the topics. The results showed that older adults responded differently based on age, gender, education and living locations (all P<0.05) when it comes to topics that of interests. The top five topics of concerned were: lifelong learning; recreation and assistive devices, nutrition and health, exercise and legal protection. The paper concluded by suggesting ways to let older adults willing to learn and take actions on various aging-in-place topics among the community-based sites.

